# Shorter leukocyte telomere length is associated with adverse COVID-19 outcomes: A cohort study in UK Biobank

**DOI:** 10.1016/j.ebiom.2021.103485

**Published:** 2021-07-23

**Authors:** Qingning Wang, Veryan Codd, Zahra Raisi-Estabragh, Crispin Musicha, Vasiliki Bountziouka, Stephen Kaptoge, Elias Allara, Emanuele Di Angelantonio, Adam S. Butterworth, Angela M. Wood, John R. Thompson, Steffen E Petersen, Nicholas C. Harvey, John N. Danesh, Nilesh J. Samani, Christopher P. Nelson

**Affiliations:** a Department of Cardiovascular Sciences, University of Leicester, Leicester, United Kingdom; b NIHR Leicester Biomedical Research Centre, Glenfield Hospital, Leicester, United Kingdom; c William Harvey Research Institute, NIHR Barts Biomedical Research Centre, Queen Mary University of London, Charterhouse Square, London EC1M6BQ, United Kingdom; d Barts Heart Centre, St Bartholomew's Hospital, Barts Health NHS Trust, London EC1A7BE, United Kingdom; e British Heart Foundation Cardiovascular Epidemiology Unit, Department of Public Health and Primary Care, University of Cambridge, Cambridge, United Kingdom; f National Institute for Health Research Blood and Transplant Research Unit in Donor Health and Genomics, University of Cambridge, Cambridge, United Kingdom; g British Heart Foundation Centre of Research Excellence, University of Cambridge, Cambridge, United Kingdom; h Health Data Research UK Cambridge, Wellcome Genome Campus and University of Cambridge, Cambridge, United Kingdom; i Medical Research Council Biostatistics Unit, Cambridge Institute of Public Health, University of Cambridge, Cambridge, United Kingdom; j The Alan Turing Institute, London, United Kingdom; k Department of Health Sciences, University of Leicester, United Kingdom; l MRC Lifecourse Epidemiology Unit, University of Southampton, Southampton, United Kingdom; m NIHR Southampton Biomedical Research Centre, University of Southampton and University Hospital Southampton NHS Foundation Trust, Southampton, United Kingdom; n Department of Human Genetics, Wellcome Sanger Institute, Hinxton, United Kingdom; o Department of Cardiovascular Sciences, University of Leicester, Glenfield Hospital, Leicester, United Kingdom

## Abstract

Background Older age is the most powerful risk factor for adverse coronavirus disease-19 (COVID-19) outcomes. It is uncertain whether leucocyte telomere length (LTL), previously proposed as a marker of biological age, is also associated with COVID-19 outcomes.

Methods We associated LTL values obtained from participants recruited into UK Biobank (UKB) during 2006–2010 with adverse COVID-19 outcomes recorded by 30 November 2020, defined as a composite of any of the following: hospital admission, need for critical care, respiratory support, or mortality. Using information on 130 LTL-associated genetic variants, we conducted exploratory Mendelian randomisation (MR) analyses in UKB to evaluate whether observational associations might reflect cause-and-effect relationships.

Findings Of 6775 participants in UKB who tested positive for infection with SARS-CoV-2 in the community, there were 914 (13.5%) with adverse COVID-19 outcomes. The odds ratio (OR) for adverse COVID-19 outcomes was 1·17 (95% CI 1·05–1·30; *P* = 0·004) per 1-SD shorter usual LTL, after adjustment for age, sex and ethnicity. Similar ORs were observed in analyses that: adjusted for additional risk factors; disaggregated the composite outcome and reduced the scope for selection or collider bias. In MR analyses, the OR for adverse COVID-19 outcomes was directionally concordant but non-significant.

Interpretation Shorter LTL is associated with higher risk of adverse COVID-19 outcomes, independent of several major risk factors for COVID-19 including age. Further data are needed to determine whether this association reflects causality.

Funding UK Medical Research Council, Biotechnology and Biological Sciences Research Council and British Heart Foundation.

**Research in context**

**Evidence before this study**

We searched PubMed for articles with the terms “telomere length” and “COVID-19” to identify publications relating to telomere length and COVID-19 outcomes. We retained only research articles that used telomere length as a biomarker, identifying three articles. All of these studies showed a relationship between shorter telomere length and either COVID-19 severity or outcome. However, all had measured leucocyte telomere length (TL) after SARS-CoV-2 infection making it difficult to interpret whether shorter TL preceded infection or was due to higher white cell turnover in response to infection.

**Added value of this study**

Our study is the largest study to date on the association of inter-individual variation in leucocyte telomere length with adverse outcomes from COVID-19 and the first to analyse telomere length that was measured prior to SARS-CoV-2 infection.

**Implications of all the available evidence**

Our study suggests that independently of age, leucocyte telomere length is associated with greater risk of poor outcome from COVID-19, possibly through an effect on immune cell senescence, and that this explains some of the heterogeneity in inter-individual response to the infection.

Alt-text: Unlabelled box

## 1 Introduction

Older age has emerged as the most powerful risk factor for severe infection, requiring hospitalisation or critical care, and mortality from coronavirus disease 19 (COVID-19) caused by severe acute respiratory syndrome coronavirus 2 (SARS-CoV-2) [1,2]. One potential mediator of this effect is ageing of the immune system, leading to increased levels of pro-inflammatory senescent cells and reduced proliferative capacity of immune precursor cells [3,4]. Telomere length (TL) is a key determinant of proliferative capacity and cellular lifespan, triggering senescence once a critically short TL is reached [5]. TL − commonly measured in leucocytes (LTL) − shows a consistent negative association with age in cross sectional population cohorts and has previously been proposed as a marker of biological age for an individual. However, age only accounts for a small proportion of the substantial inter-individual variation in LTL [6] that exists at all ages, including birth [7]. More recently, TL has also been proposed as a marker of replicative capacity and repair ability [8], both of which, within the haematopoietic system, could potentially impair an individual's response to SARS-CoV-2 infection, above any effect of age [9,10].

A few small case-control studies, in which LTL was measured after SARS-CoV-2 infection at the time of hospital admission, have reported associations of shorter LTL with hospitalisation and severe outcomes [11], [12], [13]. However, their interpretation is complicated by the possibility that LTL measurements could have been influenced by white cell turnover in response to infection. To our knowledge, no study to date has reported on associations of prior (pre-infection) LTL values and adverse COVID-19 outcomes.

Here, we examine whether LTL measured several years prior to SARS-CoV-2 infection is associated with adverse COVID-19 outcomes, leveraging our recent completion of LTL measurements in 474,074 participants aged 40–69 at time of recruitment into UK Biobank (UKB) [6] between 2006 and 2010 [14,15].

## 2 Methods

*Participants*: Participants in UKB have been characterised in detail using questionnaires, physical measurements, urinary and plasma biomarker measurements, genomic assays and longitudinal linkage with multiple health record systems, including Hospital Episode Statistics (HES) and Office for National Statistics (ONS) mortality data [16]. We have described the associations of inter-individual variation in LTL with multiple biomedical traits and risk of several diseases in UKB [15]. Since the onset of the COVID-19 pandemic, UKB has also linked participants with results from clinically indicated SARS-CoV-2 testing and COVID-19 outcomes. By linking participants in UKB to SARS-CoV-2 testing datasets of Public Health England (PHE),[17] we identified participants who tested positive between 16 March 2020 and 30 November 2020; the latter date corresponds to the latest release of HES data to UKB. We used HES records to identify SARS-CoV-2 positive participants who were admitted to hospital due to COVID-19 (ICD-10 code ‘U07.1’) within 28 days after a positive SARS-CoV-2 test. We further extracted information on need for critical care admission and respiratory support, due to COVID-19 (ICD-10 code ‘U07.1’), via linkage to the ICNARC (Intensive Care National Audit and Research Centre) database, and deaths due to COVID-19 (ICD-10 code ‘U07.1’), from the Office for National Statistics (ONS) death registry data.

The UK Biobank has ethical approval from the North West Centre for Research Ethics Committee (Application 11/NW/0382), which covers the UK. UK Biobank obtained informed consent from all participants. Full details can be found at https://www.ukbiobank.ac.uk/learn-more-about-uk-biobank/about-us/ethics. The generation and use of the data presented in this paper was approved by the UK Biobank access committee under UK Biobank application number 6077.

*LTL measurements*: Full details of the LTL measurements in UKB are provided elsewhere [6]. Briefly, LTL was measured using an established PCR method that expresses LTL as a ratio (T/S ratio) [6]. LTL measurements were adjusted for technical variation, log_e_ transformed and Z-standardised [6]. In order to assess and adjust for within individual variability in LTL we measured LTL at two time-points (mean interval: 5·5 years) for 1351 participants, yielding a regression-dilution ratio of ~0·68. Results in this study have been corrected for within-person variability of LTL values over time (abbreviated "usual LTL"), as described previously [6,15].

*Outcome definitions:* Our study's primary outcome was a composite of COVID-19-related outcomes (ICD-10 code ‘U07.1’): hospital admission, requirement for critical care, respiratory support, or mortality. We defined cases as those participants in UKB who tested positive for SARS-CoV-2 and had the primary outcome. For our primary outcome, controls were those who tested positive for SARS-CoV-2 but were not hospitalised within 28 days. To reduce the scope for collider bias [18] we included only participants with positive SARS-CoV-2 tests done outside of hospital settings, since hospital admission itself may increase the likelihood of SARS-CoV-2 testing. The age, sex and ethnicity adjusted odds ratio (OR) for having a SARS-CoV-2 test (*n* = 43,574) at any location, was 1·03, (95% CI 1·01-1·05; logistic regression *P* = 1·0 × 10^−4^) per 1-SD shorter usual LTL.

We conducted several secondary analyses. First, we examined associations with each component of the primary composite endpoint. Second, we analysed the primary outcome using the rest of UKB participants as controls, as testing was unlikely to be random and the restriction to SARS-CoV-2 positive controls only is potentially subject to selection bias related to factors associated with infection [19]. Third, to ensure that apparently post-COVID-19 outcomes were not re-admissions or influenced by proximate medical events prior to infection, we excluded participants with any hospital admission in the previous 6 months. Finally, we consider the impact of inflammation and baseline disease prevalence on LTL, to minimise the potential confounding from these factors on any LTL-COVID-19 outcomes relationship.

*Statistical analysis:* Univariable tests were performed using T-tests for continuous traits and Fishers exact or χ^2^ tests for categorical traits as appropriate. Analyses involved multivariable logistic regression, adjusting for age (at SARS-CoV-2 positive test), sex and ethnicity. Due to small numbers, ethnic groups other than White were combined and participants with missing ethnicity (*n* = 14 cases and 46 controls) were excluded. To remove the correlation between LTL and age, we used the residuals of LTL adjusted for age at baseline within the statistical models. In specific models to estimate the impact on the association due to inflammation or disease prevalence we re-estimated LTL residuals adjusting for age at baseline and C-reactive protein or any of 123 curated diseases [15] (Supplementary Table), respectively. ORs were further adjusted for baseline smoking status and body-mass index (BMI) recorded at entry into UKB. Results are described as ORs associated with the outcome per one standard deviation (SD) shorter LTL residual, with associated 95% confidence intervals (CI) and *p*-values.

In an exploratory analysis, we conducted one-sample Mendelian randomisation (MR) analyses in UKB [20] to evaluate a causal relationship between shorter LTL and adverse COVID-19 outcomes, using the inverse-variance weighted (IVW) [21] and weighted median [22] methods with a set of 130 genome-wide significant (*P* < 8.31 × 10^−9^), conditionally independent, uncorrelated and non-pleiotropic genetic variants we recently identified as genetic instruments for LTL [15]. We used MR-Egger regression to assess robustness to horizontal pleiotropy [23].

*Role of the funding body*: The Funders had no role in study design, data collection, data analyses, interpretation, or writing of this study.

## 3 Results

By 30 November 2020, 914 participants were identified with an adverse COVID-19 related outcome and 5861 participants were identified as primary controls (positive community test for COVID-19 but not hospitalised). Their characteristics are summarised in Table 1. On average, compared to controls, cases were older and more likely to be male and from a non-White background. At time of their entry into UKB, they also had a higher BMI and more likely to be current smokers. (Table 1).

**Table 1:** Characteristics of participants by case status.

**Trait**		**Cases *N*** **=** **914**	**Controls *N*** **=** **5861**	***P*-value**
**Age at COVID-19 test**		70 (8·00)	64 (8·00)	1·85E-95
**BMI**		29·61 (5·39)	27·97 (4·86)	1·55E-20
**Sex**	**Male**	573 (62·69)	2675 (45·64)	8·20E-22
	**Female**	341 (37·31)	3186 (54·36)	
**Ethnicity**	**Asian**	33 (3·61)	217 (3·70)	0·006
	**Black**	5 (0·55)	38 (0·65)	
	**Chinese**	3 (0·33)	9 (0·15)	
	**Mixed**	36 (3·94)	117 (2·00)	
	**Other**	8 (0·88)	78 (1·33)	
	**White**	829 (90·70)	5402 (92·17)	
**Smoking**	**Never**	358 (39·43)	3236 (55·38)	1.27E-18
	**Ex-smoker**	408 (44·93)	2001 (34·25)	
	**Current**	142 (15·64)	606 (10·37)	
**LTL (age adjusted)**		-0·14 (0.97)	-0·03 (1·00)	0·002

Data shown are mean (SD) for continuous traits or n (%) for categorical traits. LTL, smoking status, BMI, sex and ethnicity are from baseline information. LTL is log-transformed and Z-standardised. P-values were obtained via t-tests for continuous traits, Ethnicity was assessed using Fishers exact test and other categorical traits were tested using a χ^2^ test.

LTL at entry to UKB was on average shorter in cases compared with controls (Table 1). The OR for the primary outcome was 1·17 (95% CI 1·05-1·30; logistic regression *P* = 0·004) per 1-SD shorter usual LTL, after adjustment for age, sex and ethnicity (Table 2). The OR only slightly attenuated after further adjustment for smoking status and BMI (OR = 1·15, 1·03-1·28), and after adjustment for the presence of any of 123 diseases recorded at baseline (OR = 1·14, 1·02-1·26), while adjusting for CRP slightly increased the estimated effect size (OR = 1·19, 1·06-1·33). As expected, older age, male sex and non-White ethnicity were each associated with higher risk of adverse COVID-19 outcomes independently of usual LTL (Table 2).

**Table 2:** Results of the main and secondary/sensitivity analyses.

	***N* cases**	***N* controls**	**Odds Ratio (95%CI)**	***P*-value**
**Composite outcome**				
LTL (age-adjusted) (per 1 SD shorter)	914	5861	1·17 (1·05, 1·30)	0·004
Age at COVID-19 test (per 5 yrs older)			1·58 (1·51, 1·65)	<0·001
Sex (male vs female)			1·88 (1·62, 2·19)	<0·001
Ethnicity (non-White vs White)			1·80 (1·39, 2·34)	<0·001
Separate components as outcome*				
Hospitalisation	672	5861	1·17 (1·03, 1·32)	0·013
Critical care support	383		1·31 (1·12, 1·53)	<0·001
Respiratory support	279		1·36 (1·13, 1·64)	<0·001
Death	157		1·36 (1·07, 1·72)	0·013
**Population controls**				
LTL (age-adjusted) (per 1 SD shorter)	914	465,946	1·19 (1·08, 1·31)	<0·001
**Excluding participants with recent hospitalisation**				
LTL (age-adjusted) (per 1 SD shorter)	732	5861	1·15 (1·02, 1·30)	0·019
**Mendelian Randomisation**				
MR IVW	914	5861	1·30 (0·85, 2·00)	0·224
MR-median			1·25 (0·62, 2·50)	0·537

The main analysis is based on our composite outcome and the full multivariable model estimates are shown for each risk factor. *For each component of the composite outcome analysed separately, the results shown for these are labelled by outcome component but represent the LTL (age-adjusted) estimate (per 1 SD shorter). For each analysis, the numbers of cases and controls are given alongside the odds ratio, 95% confidence interval and *P*-value (from logistic regression models or MR). MR IVW: Mendelian randomisation inverse-variance weighted method. MR-median: Mendelian randomisation weighted median method.

**Fig. 1:**
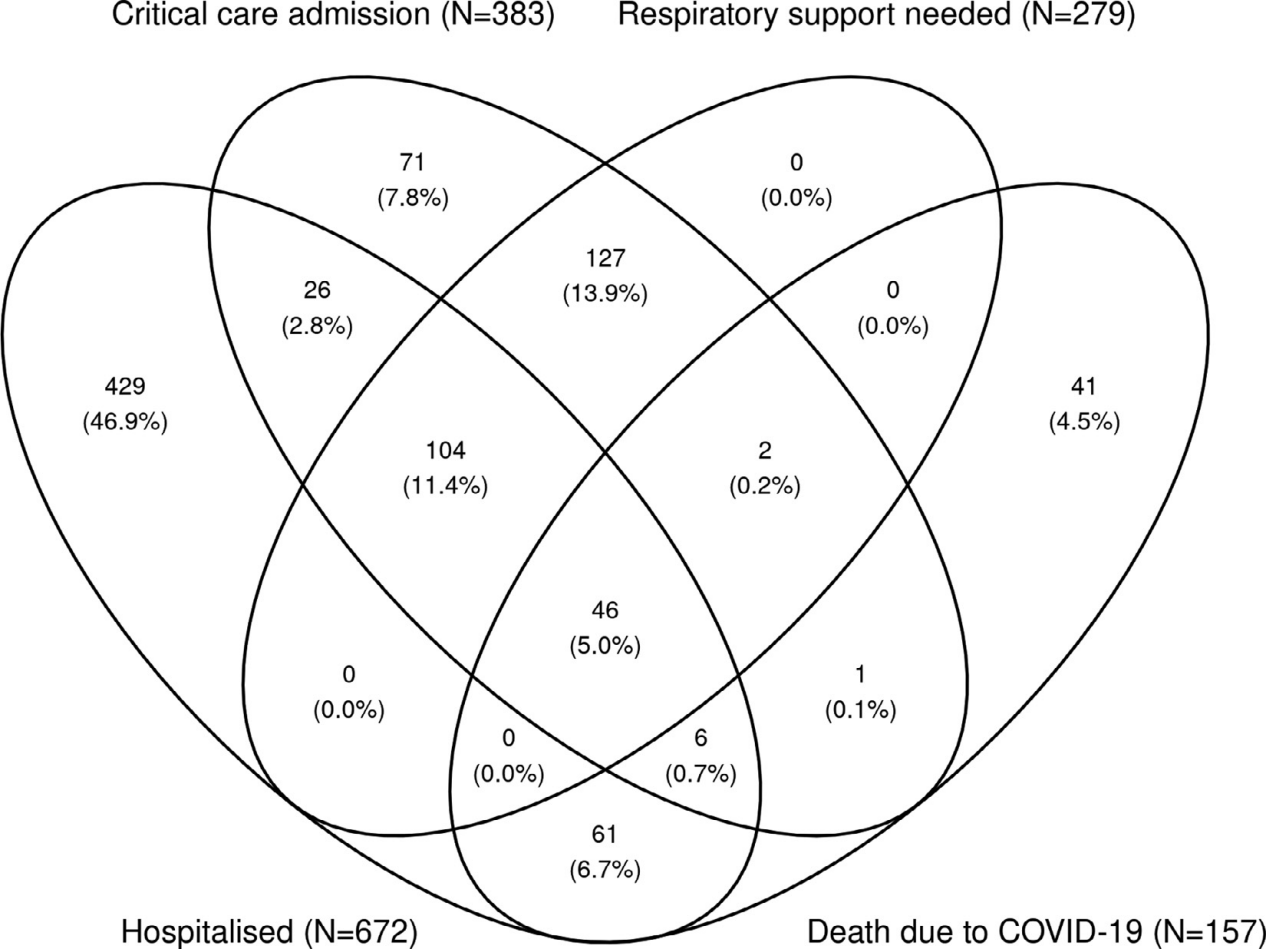
Venn diagram showing the distribution of the individual components of the primary outcome. Where *N* is the frequency and: Hospitalised, due to COVID-19; Critical care admission, due to COVID-19; respiratory support needed, while in critical care due to COVID-19; death due to COVID-19.

Sub-components of our study's primary composite outcome were not mutually exclusive, as 46 cases contributed to all four sub-components (Fig. 1). Shorter usual LTL was significantly associated with higher risk of each sub-component (Table 2). ORs were broadly similar to the main findings in analyses that replaced the SARS-CoV-2-positive control group with all UKB participants as controls or that excluded any participant with a hospital admission in the six months prior to testing positive for SARS-CoV-2 (Table 2).

In MR analyses, the IVW odds ratio was 1·30 (0·85–2·00; MR-IVW *P* = 0·224) per 1-SD shorter genetically-determined LTL, a non-significant result directionally concordant with the observational finding (Table 2). Results were similar using the weighted median method (Table 2) and there was no evidence of horizontal pleiotropy (MR-Egger intercept *P* = 0·591).

## 4 Discussion

In a study of 6,775 participants with a positive test for SARS-CoV-2 (nested within the 500,000-participant UKB), we have shown that individuals with shorter LTL assessed several years *prior* to SARS-CoV-2 infection had higher risk of adverse COVID-19 outcomes, even after adjustment for several established risk factors for COVID-19 including age. This finding suggests that shorter LTL is likely to be independently associated with COVID-19 hospitalisation and severity. The results of analysis of LTL-associated genetic variants and COVID-19 were directionally concordant with our observational findings but non-significant. Our results, therefore, encourage further investigation of the potential causal relevance of TL to adverse COVID-19 outcomes.

The validity of our results is supported by several observations. First, our study found associations between older age, male sex, and non-White ethnicity that have previously been linked with adverse COVID-19 outcomes in the UK [2]. Each of these factors was associated much more strongly with COVID-19 outcomes than was shorter LTL. Second, we found significant associations of shorter LTL with each sub-component of our study's primary composite outcome. Third, our main findings persisted after adjustment for multiple risk factors. Fourth, our overall result was robust to sensitivity analyses designed to minimise the scope for potential biases. For example, collider bias can lead to false associations between a risk factor and an outcome, [18] as highlighted by studies related to understanding of COVID-19 disease risk and severity [19]. Indeed, we found evidence for potential colliders in our own analysis, observing a small but significant association between shorter LTL and higher likelihood of SARS-CoV-2 testing. Hence, we only included participants with a positive SARS-CoV-2 test outside the hospital setting, as hospitalisation itself may increase the likelihood of testing. Finally, considering the potential impact of inflammageing on the observed result we further adjusted for C-reactive protein and found no meaningful changes in the association.

The biological mechanisms through which shorter LTL might increase risk of adverse outcomes from SARS-CoV-2 infection remain to be clarified. Our finding that the association was not substantially attenuated when we adjusted for the association of LTL with multiple diseases at baseline, suggest that, if this association is causal, it is probably not simply a reflection of co-morbidity due to the impact of shorter LTL on risk of these diseases. A potential mechanism relates to the impact of telomere length dynamics on aging of the immune system [24] and the potential role of senescence in severe SARS-CoV-2 infection [3,4,25]. While we have measured TL in leucocytes we believe these results likely reflects TL within T-cells in this scenario, although further studies would be required to confirm this. When challenged with infection, individuals with shorter LTL prior to infection would potentially have less proliferative capacity within the T-cell population required for an efficient response to SARS-CoV2, coupled with reduced lymphopoiesis following infection [9,26]. Individuals with shorter LTL may also potentially already harbour a higher proportion of senescent T-cells, reducing the number of functional cells that are able to respond to infection [25]. Additionally, senescent cells are known to adopt a pro-inflammatory phenotype, secreting high levels of cytokines, which can further drive inflammation in COVID-19 patients [25]. Our results are also in concordance with previous studies showing that shorter LTL increases the risk of adverse outcome in other infections [27,28].

Our study has several limitations. UKB is not representative of the general UK population; only 6% of those invited to participate did so [29]. The age distribution within UK Biobank includes participants aged 40–70 at baseline, who will be about 10–15 years older now, limiting the ability to assess association in individuals in other age groups. We were unable to fully assess ethnicity due to small numbers, though over time this limitation can be resolved with increased case numbers. Risk factor levels and mortality rates are lower than in the general population, although risk factor associations with mortality for a range of diseases are similar [30]. Hence, further studies are warranted in other populations. Our one-sample Mendelian randomisation analysis in UKB had limited power to reliably estimate causal effects as fewer than one thousand participants had been hospitalised after a positive SARS-CoV-2 test and our genetic instrument of 130 variants, while using the most up to date information on LTL-associated variants, accounts for only ~4% of inter-individual variation in LTL [15]. While there are data from large genetic studies of COVID-19 [31], they could not be used in our analysis because the outcome definitions differed substantially from those we used, and because of their inclusion of within hospital testing that is potentially a collider with LTL and COVID-19 outcomes. Larger sample sizes with comparable disease phenotypes should, therefore, enable more precise evaluation of a potential causal association between shorter LTL and adverse COVID-19 outcomes.

In conclusion, in the largest study to date, we provide evidence that shorter LTL is associated with higher risk of adverse COVID-19 outcomes, independent of several major risk factors for COVID-19.

## Data sharing

Source data is accessible via application to the UK Biobank.

## Author Contributions

V.C., C.P.N., J.N.D, S.E.P, N.C.H. and N.J.S conceived the project. All authors contributed to the sample definition and the analysis plan. Q.W., C.M. and C.P.N. performed the analyses. V.C., C.P.N., Q.W. and N.J.S. prepared the manuscript and all authors revised it. V.C., C.P.N., J.R.T., J.N.D. and N.J.S. (Principal investigator) secured funding and oversaw the project.

## Declaration of Competing Interest

These authors declare no competing interests.

## Appendix Supplementary materials

Image, application 1
